# IDIOPATHIC ERUPTIVE MACULAR PIGMENTATION: REPORT ON TWO CASES

**DOI:** 10.4103/0019-5154.70698

**Published:** 2010

**Authors:** Sanjiv Grover, Atoshi Basu

**Affiliations:** *From the Department of Dermatology, Armed Forces Medical College, Pune, Maharashtra, India*

**Keywords:** *Idiopathic*, *eruptive*, *macular pigmentation*

## Abstract

Idiopathic eruptive macular pigmentation (IEMP) is a rather under-reported condition of unknown etiology. Clinically consisting of benign hyperpigmented macules, the condition is characterized histopathologically by dermal melanization. It must be differentiated from lichen planus pigmentosus, erythema dyschromicum perstans, fixed drug eruption and mastocytosis.

## Introduction

*I*diopathic *E*ruptive *M*acular *P*igmentation (IEMP) is a rather under-reported condition of unknown etiology first reported three decades ago.[1] Awareness of its existence is low and details of its clinico-pathological features are not very well known. We report two such cases of this little known condition.

## Case Report

**Figure 1:**
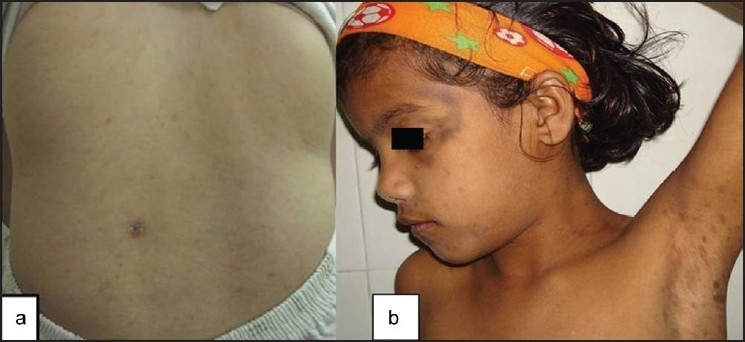
Multiple, discrete, ill-defined, gray-black pigmented macules on trunk [Panel a], face and limbs [Panel b]

**Figure 2:**
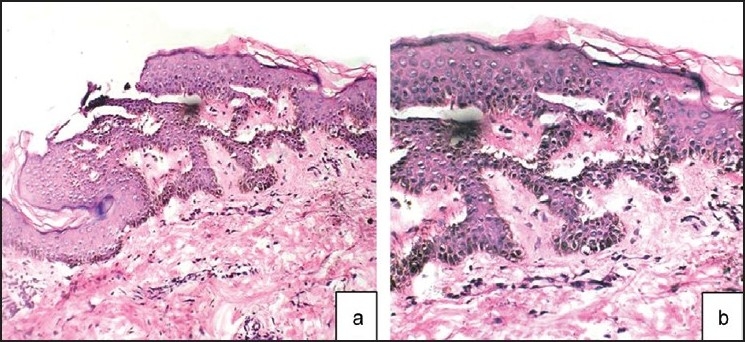
Mild papillomatosis, increased basal layer melanin and dermal melanophages [H and E; ×100 (Panel a), ×400 (Panel b)]:

The details of the cases are illustrated in Table 1. Both the cases were asymptomatic; the lesions appearing insidiously and progressing gradually to involve the affected areas. There was no history of inflammatory lesions or topical applications predating the onset of lesions, systemic drug intake or itching. There was no personal or family history of atopy and the antenatal history was unremarkable. General physical and systemic examination was normal. Dermatological examination revealed multiple, discrete, ill-defined, gray-black pigmented, smooth, non-scaly, non-atrophic, non-alopecic, normesthetic, oval macules measuring app 5mm to 15 mm in size over the involved areas [Figure 1]. Darier’s sign was negative. Hair, nails, mucosae, palms and soles were normal. Hematological and biochemical investigations were normal. Histopathology of the hyper pigmented macule revealed mild papillomatosis, increased basal layer melanin and dermal melanophages. There was absence of parakeratosis and dermal inflammatory/lichenoid infiltrate [Figure 2].

**Table 1:** Brief clinical summary of cases

Sex/Age	Lesions	Site	Duration
F/13	Ill-defined, grayblack macules	Trunk, proximal upper and proximal lower limbs	8 months
F/07	Ill-defined, darker, gray-black macules	Face, neck, proximal upper limbs, trunk	4 months

## Discussion

Less than 30 cases of IEMP have been reported in literature so far, reflecting unfamiliarity with the entity. While majority of the cases have been reported in children and adolescents, it has also been reported in a 31-year-old female.[2] Criteria have been established for diagnosis of IEMP,[3] namely: (a) eruption of brownish, non-confluent, asymptomatic macules involving the trunk, neck and proximal extremities in children and adolescents (b) absence of preceding inflammatory lesions (c) no previous drug exposure (d) basal layer hyperpigmentation of the epidermis and prominent dermal melanophages without visible basal layer damage or lichenoid inflammatory infiltrate (e) normal mast cell count. Our cases fulfilled all these criteria for diagnosis of the entity.

IEMP must be differentiated from lichen planus pigmentosus, erythema dyschromicum perstans, fixed drug eruption and mastocytosis. Absence of basal cell degeneration, colloid bodies, lichenoid infiltrate or excess dermal mast cell population may rule out these disorders. In a series of nine cases, an additional histopathological finding of pigmented papillomatosis has been reported.[4] However, in this series, some lesions were reported to have clinically a velvety surface and these correlated with the peculiar histopathological finding. Otherwise, IEMP is an epidermal hypermelanotic condition with basal cell melanization. The condition is self-limiting and has been reported to disappear spontaneously in months to years.[5] However, a solitary report of a case of IEMP has been reported for 21 years duration with one recurrence.[6]

The cases are reported for rarity in literature and to underscore the clinical suspicion of this entity in differential diagnosis of pigmented lesions, especially in the first two decades of life.
